# The Symbiotic Fungus *Leucoagaricus gongylophorus* (Möller) Singer (Agaricales, Agaricaceae) as a Target Organism to Control Leaf-Cutting Ants

**DOI:** 10.3390/insects13040359

**Published:** 2022-04-06

**Authors:** Sean Araújo, Janaína Seibert, Ana Ruani, Ricardo Alcántara-de la Cruz, Artur Cruz, Alana Pereira, Doraí Zandonai, Moacir Forim, Maria Fátima Silva, Odair Bueno, João Fernandes

**Affiliations:** 1 Chemistry Department, Sao Carlos Federal University, UFSCar, São Carlos 13565-905, Brazil; seansaraujo@hotmail.com (S.A.); jana_seibert@hotmail.com (J.S.); anapruani@gmail.com (A.R.); arturdasilvacruz@yahoo.com.br (A.C.); alanakelyene@gmail.com (A.P.); dorai@ufscar.br (D.Z.); mrforim@ufscar.br (M.F.); dmfs@ufscar.br (M.F.S.); 2 Centro de Ciências da Natureza, Campus Lagoa do Sino, Sao Carlos Federal University, Buri 18290-000, Brazil; ricardo.cruz@ufscar.br; 3 Laboratório de Biologia Química Microbiana (LaBioQuiMi), Institute of Chemistry, Universidade Estadual de Campinas (UNICAMP), Campinas 13083-970, Brazil; 4 Center of Study of Social Insects, Department of Biology, Campus Rio Claro, Sao Paulo State University (UNESP), Rio Claro 13506-900, Brazil; odair.bueno@unesp.br

**Keywords:** pesticide, antifungal activity, chemical control, biological control, natural products, synthetic compound

## Abstract

**Simple Summary:**

The most used approach to control leaf-cutting ants (which cause damage in agricultural areas) is the application of synthetic chemical compounds that directly affect these insects. But another approach is the use of natural substances that attack the symbiotic fungus responsible for many aspects of the survival of the nest. In this study, we discuss the natural substances already reported in the literature to have fungicidal activity and how they could be applicable as products for the control of leaf-cutting ants.

**Abstract:**

*Atta* and *Acromyrmex* are the main genera of leaf-cutting ants present in North and South America, causing extensive damage to agroforestry. Control of the ants requires high handling costs with few effective methods available to decrease the losses. The symbiosis between the leaf-cutting ants and the fungus *Leucoagaricus gongylophorus* is essential for ant nest survival. Therefore, *L. gongylophorus* may be a key target in controlling leaf-cutting ants, since its reduction may cause an imbalance in the symbiosis necessary to maintain the nest. Among the options for natural fungal control, plant species are considered important sources of compounds belonging to several classes of natural products that show potential as antifungal agents. This review also presents studies that establish that the antagonist fungi from the *Escovopsis* and *Trichoderma* genera effectively reduce the development of *L. gongylophorus*. The development of nanostructured delivery systems, which have shown advantages over conventional formulations, is suggested for ant control; no commercial nanotechnology-based product has yet been developed, and this appears to be a new approach for future studies.

## 1. Introduction

Large-scale insect populations that damage crops are considered pests and include *Sitophilus zeamais* Motschulsky (Coleoptera: Curculionidae), the maize weevil, which impairs the storage of corn grains (*Zea mays*, Poales: Poaceae) with a large occurrence in Brazil [1]; *Spodoptera frugiperda* Smith (Lepidoptera: Noctuidae), the fall armyworm, which causes damage to soybean (*Glycine max*, Fabales: Fabaceae), sorghum (*Sorghum bicolor*, Poales: Poaceae), sugar cane (*Saccharum officinarum*, Poales: Poaceae), etc. and is native to the tropical regions of North and South America [2,3]; *Plutella xylostella* L. (Lepidoptera: Plutellidae), the diamondback moth, the main pest in the cultivation of cabbage (*Brassica oleracea* var. capitata, Brassicales: Brassicaceae) and cauliflower (*B. oleracea* var. botrytis, Brassicales: Brassicaceae), it has an extensive distribution in tropical and subtropical areas [4,5]; and ants of genera *Atta* and *Acromyrmex* (Formicidae: Attini), which attack a great diversity of plants [6].

The fungus *Leucoagaricus gongylophorus* (Möller) Singer (Agaricales, Agaricaceae) is the symbiotic partner of several species of Attine ants, whose development on leaves cuts inside the nests to form the fungus gardens. The fungus produces enzymes that degrade leaf polysaccharides into nutrients assimilable by ants. Among these nutrients, glucose produced from plant material in the fungus garden appears to be the main source of food for ants along with proteins and amino acids [7]. Leaf-cutting ants from the Myrmicinae family, tribe Attini (Hymenoptera: Formicidae), are native to the Neotropics and belong to two genera: *Atta* and *Acromyrmex* [6,8,9].

The use of chemical pesticides for insect pest control, often applied without legal supervision, has led to environmental issues such as the death of beneficial insects, parasitoids, and predators, and pesticide residues in the soil [10]. Studies aimed at discovering active compounds from natural sources have contributed to the identification of new agrochemical substances [11].

The characterization of plant extracts and natural compounds that have shown direct lethal activity on leaf-cutting ants is well explored, such as the extracts of *Asclepias curassavica* (Asclepidaceae) [12] and *Virola sebifera* (Myristicaceae) [13] and isolated compounds such as alkaloids, limonoids [14], sesquiterpenes [15], and flavonoids [16].

Another approach to control leaf-cutting ants would be the application of extracts or compounds acting on the symbiotic fungus *L. gongylophorus*, an important microorganism in the management and survival of the nest [14,16,17]. Based on a mutualistic relationship with the leaf-cutting ants, the control of this insect can be carried out at the insecticide or fungicide level, either individually or by combining these two strategies to provide an efficient integrated control [18,19]. Baits with insecticides such as chlorpyrifos, deltamethrin, fipronil, and permethrin, but mainly sulfluramid, based on citrus pulp on which the mutualistic fungus feed, have been the main tool for the control of leaf-cutting ants [20,21]. Sulfluramid is one of the most successful active ingredients for the control of leaf-cutting ants because it is chemically and physically stable and has a delayed toxic action [22,23]. In addition, these eusocial insects do not detect it as a hazardous material, that is, sulfluramid is not repellent and is distributed by the ants themselves in a large number of fungus garden compartments inside the nest contaminating as many ants as possible when they feed [24]. However, sulfluramid was listed as a persistent organic pollutant at the Stockholm Convention in 2015 [25,26]. Therefore, it is necessary to find alternatives to sulfluramid for the control of leaf-cutting ants, including methods or active compounds that act on the mutualistic fungus; however, this control approach has been little explored and there are no baits with fungicide that act directly on *L. gongylophorus*.

Products used in pest control, in addition to being effective, should be safe for the environment and non-target organisms. Thus, the use of alternative methods involving natural substances or based on them (synthetic compounds), as well as the use of other microbes with fungicidal action, is convenient. In this context, the present review reports examples of chemical and biological controls that have shown potential against the symbiotic fungus *L. gongylophorus* and that could be better explored.

### 1.1. Damage Caused by Leaf-Cutting Ants

Leaf-cutting ants are eusocial insects, as they show a highly developed social structure, manifesting ecological relationships [27]. Their complex structure is characterized by an organized social behavior, the cultivation of a fungus garden and high levels of hygiene, which hinders the management of leaf-cutting ants compared to other insects [23].

Leaf-cutting ants cause damage in agricultural and silviculture areas, mainly in monocultures. Plants aged 1 to 3 years are the main targets of these ants and are defoliated, causing irreversible damage, since the seedlings are new and fragile [28], causing significant yield losses [29,30]. Defoliation affects the growth of plants because the increase in diameter is dependent on the current level of photosynthesis, which is reduced by the loss of leaves [31]. In addition, a lesser effect is observed on height loss, since growth is related to the plant reserves.

A study carried out in *Pinus taeda* plantations in Argentina concluded that there was a reduction of 17.3% in the growth in the neck diameter of plants aged up to 12 months attacked by leaf-cutting ants belonging to the genus *Acromyrmex* [32]. In another study carried out in 10-year-old *Pinus caribaea* trees in Venezuela, attacks by *Atta laevigata* reduced wood production up to 50% ha^−1^ [33]. In this same way, a level of economic damage between 13.4 and 39.2 m^2^ ha^−1^ was observed in a eucalyptus site in areas of the Atlantic Forest after infestation by leaf-cutting ants (*Atta* sp.) [34]. Similar values (7.02 to 34.86 m^2^ ha^−1^) were also observed for the same cultivation in Cerrado, another Brazilian biome [26].

Leaf-cutting ants (Hymenoptera: Formicidae) of the genera *Atta* and *Acromyrmex* are the main pests in Brazil, affecting *Pinus* and *Eucalyptus* plantations [35]. These leaf-cutting ants removed 20–30% of the total leaf area of fruit, cocoa, and crops of other plants documented in Trinidad and in Guadalupe (Central America) [36], resulting in an annual loss higher than USD 250,000. Measurements of four *Eucalyptus* stands showed a total of 2327 nests of leaf-cutting ants with 4742.27 m^2^ of surface area (loose soil). The number of leaf-cutting ant nests and their area per hectare were 64.52 and 238.90 m^2^, respectively [37]. Between 1991 and 1996, the average total cost of combatting attacks by leaf-cutting ants in a 6-month-old eucalyptus forest was USD 12.60 per ha^−1^ in Brazil [38]. In 2011, the updated values for the control of this pest in Brazil were around USD 1.23 per ha^−1^, considering a market value of eucalyptus wood of USD 18.00 per m^−3^ [34,37].

Due to the vast regions affected by leaf-cutting ants, avoiding the damage they cause requires high control costs. Economic losses from leaf-cutting ants, either through reduced yields or through expenses for their control, run into the billions of dollars worldwide, reaching more than 30% of the total costs spent on forest management [39]. Fenitrothion, fipronil, or sulfluramid are examples of the most common synthetic active ingredients used to combat leaf-cutting ants, but they are banned in some countries [36]. Although other methods have been evaluated, these chemicals are the only ones to show really satisfactory effects for controlling this pest [9,23,40]. Thus, a better understanding of how the structure of leaf-cutting ant nests works as well as the division of tasks between their mutualistic organisms are key points in the search for new targets to combat this pest.

### 1.2. Biological Relationship between Leaf-Cutting Ants and Their Mutualistic Fungus

The description of a complex microbial environment of leaf-cutting ants, their obligatory association with the basidiomycete species *L. gongylophorus,* and the maintenance of the fungus gardens is well reported in many studies [41,42,43]. Through the symbiotic relationship between leaf-cutting ants and the fungus *L. gongylophorus*, the microorganism offers enzymes that break down plant tissues and detoxify some compounds that may have insecticidal characteristics so that the ant has access to plant material that would not be possible without the work of the fungus [44,45].

Glucose produced from plant material in the fungus garden seems to be the main food source for leaf-cutting ants of the genus *Atta* [46]. *L. gongylophorus* produces glucose from plant material through, for example, starch hydrolysis by fungal extracellular α-amylase and maltase. This is an ongoing process inside the ants’ nest by which this symbiotic fungus contributes to the ant nutrition with starch [47]. Starch and xylan are consumed by *L. gongylophorus* faster compared to cellulose. This fungus can efficiently hydrolyze these polysaccharides and assimilate xylose, maltose, and glucose in order to make these nutrients available to the ants [48].

*L. gongylophorus* produces swollen structures at the tip of the hypha called gongylidia. A grouping of gongylidia forms staphylae, which provide food for the ants and their larvae [49]. The process takes place by the harvesting of a staphylum or bundle of hyphae by the workers and placing it directly in the larval mouthparts, or the workers handle the staphylae or hyphae until they reach the ideal consistency and then deposit them in the larval mouthparts [50]. Staphylae or hyphae are the main sources of nutrients for immature ants. Larvae need a high protein intake for growth, which can only be provided by *L. gongylophorus* [51]; when they are fed an artificial staphylae-based diet they gain more weight [52]. Larvae of *Atta cephalotes* obtain 100% of their feed from staphylae, but workers obtain only 4.8% of their respiratory energy needs from this source, while the rest of their needs are presumably supplied by the sap of the plant [52]. In other words, the fungus is not the greatest source of food for workers, since they take up nutrients while harvesting plant material [53].

**Figure 1:**
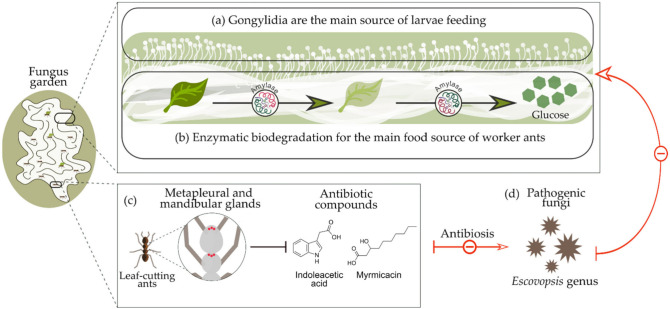
Symbiotic relationship between leaf-cutting ants and *Leucoagaricus gongylophorus* symbiotic fungus: (**a**) Gongylidia produced in the fungus garden are the main source of food for the ant larvae; (**b**) fungus enzymes such as amylase are responsible for the biodegradation of organic material into glucose, the main food source for the worker ants; (**c**) leaf-cutting ants produce antibiotic compounds that protect the fungus garden against harmful agents; (**d**) pathogenic microorganisms such *Escovopsis* fungal genera.

Workers do not digest many of the fungal enzymes when they consume gongylidia, but they deposit these enzymes in the upper parts of the fungus garden through the fecal fluid of the chewed plant material, thus providing adequate environmental conditions to nourish their fungal partners [54,55]. This allows the cultivation by ants to surpass other microbes present in the garden. For example, ants are generally known to eliminate foreign microbes within their gardens by producing antibiotics in their metapleural and mandibular glands [56,57], such as 3-hydroxydecanoic (myrmicacin) and indoleacetic acid, produced from the genera *Atta* and *Acromyrmex* [58]. A schematic summary of the relationship between leaf-cutting ants and their symbiotic fungus *L. gongylophorus* is shown in Figure 1.

## 2. Fungal Control

Different compounds have been studied as novel alternatives for the control of leaf-cutting ants, since commercially available products, generally synthetics, have disadvantages such as side effects towards non-target organisms and environmental contamination. Searches directed at Brazilian Forest Stewardship Council-certified forestry companies showed that chemical control (82%) is the most important method for pest management, followed by biological control (71.4%), tree resistance (54%), cultural control (37.5%), and mechanical control (34.5%) [59]. Based on this, a comparison between the main chemical and biological controls with effects on the fungus garden was carried out in order to propose new agents to replace the commercially synthetic available products.

“Leaf-cutting ant *”, “fungus growing ant *”, and “leaf cutter ant *” were used as query keywords in the PubMed and Web of Science databases. The use of the symbol “*” in the databases is not to limit the search only to the characters of keywords, but to search for words with similar spellings. Studies published through 31 August 2020 and written in the English, Portuguese, and Spanish languages were selected. The total search found 1929 articles (1924 in English, 1 in Portuguese, and 4 in Spanish); after a complete reading of the abstracts, only 26 articles were included in this review. Review manuscripts and studies related to chemical characterization analysis only were excluded. Extracts and isolated and synthetic compounds as well as microorganisms reported in trials against *L. gongylophorus* were tabulated and classified according to the type of control. The selected materials were considered active according to the methodology applied in each work.

### 2.1. Chemical Control

#### 2.1.1. Natural Compound

The chemical complexity of natural compounds is an important source in the search for new antifungal agents; however, some naturally occurring structures can be difficult to produce synthetically [60], but they can be used as sources of semi-synthetic compounds [61]. In addition, natural compounds can be less dangerous and persistent, which can lessen the risks compared to conventional products used in crop pest control [62]. These qualities, added to the antimicrobial potential presented by essential oils and plant extracts, reinforce the promise of these products as alternatives for agricultural use (Table 1). In this sense, several works have already demonstrated the potential of natural compounds as fungicidal agents on the symbiotic microorganism of leaf-cutting ants. Although all the studies reported here were based on in vitro methods, the results are promising and the data can be used for future trials in in vivo systems to confirm the effect on leaf-cutting ant control.

In screening plant extracts with action against *L. gongylophorus* through the bioautography assay, 11 species were reported: *Achyrocline tomentosa* (Asteraceae)*, Aristolochia argentina* (Aristolochiaceae)*, Baccharis linearifolia* (Asteraceae)*, B. coridifolia* (Compositae)*, B. flabellata* (Asteraceae)*, Dalea elegans* (Fabaceae)*, Flourensia oolepis* (Asteraceae)*, Grindelia pulchella* (Asteraceae)*, Pterocaulon alopecuroides* (Asteraceae)*, Trichocline reptans* (Asteraceae)*, and Zanthoxylum coco* (Rutaceae), with *A. argentina* and *P. alopecuroides* showing the greatest biological effect. In addition, among the *Baccharis* species, *B. flabellata* can be highlighted [63]. A reduction in fungal biomass has also been observed for extracts of *Ageratum conyzoides*, *Coriandrum sativum*, and *Mentha piperita*. Although all species were effective at a concentration of 100 mg/mL, *M. piperita* maintained the effect at the lowest concentration (25 mg/mL; Table 1) [64].

A difference in antifungal activity against *L. gongylophorus* was observed between *Capsicum baccatum* and *C. frutescens* [65], although the types of secondary metabolites detected in both were the same (Table 1). However, higher amounts of flavonoids were quantified in *C. frutescens*, suggesting that this class of compounds would be responsible for its enhanced antifungal activity. Another hypothesis would be that the volatile and alkaloid compounds are related to this antifungal activity, as caryophyllene and conhydrine were detected only for this species.

Factors intrinsic to the plant material, as well as the method and the type of solvent used to obtain the extracts, can interfere with the antifungal activity against *L. gongylophorus* presented by the same species. For example, extracts of *Sesamum indicum* were prepared in chloroform, methanol, and water. Interestingly, there were no differences in the effect on fungal growth for the first two solvents, but the aqueous extract showed no effect [8]. Considering the different types of plant parts, it was possible to observe that greater antifungal activity was found for fruit and seed extracts than for leaves (Table 1) [8]. The correct choice of plant part as well as the type of extraction solvent is crucial for the successful discovery of new bioactive substances.

Another important parameter is the correct choice of experimental model. Assessing three methodologies of antifungal activity, *Senna alata* was the most effective in controlling *L. gongylophorus,* independent of the method used, followed by *Allium sativum, Manihot esculenta, Allium cepa,* and *Lycopersicon esculentum* (Table 1) [19]. In this case, the difference in antifungal activity can be attributed to the greater levels of alkaloids in the two species with the greatest inhibitory activity.

Extracts obtained from microorganisms have also shown action against the mutualistic fungus *L. gongylophorus* (Table 2). Extracts of *Escovopsioides* and *Escovopsis* were obtained in the presence or absence of the target fungus [66]. For *Escovopsioides nivea*, no difference in the fungal growth inhibition was observed regardless of the type of extract. On the other hand, the extracts obtained from co-cultivation between *Escovopsis* strains and *L. gongylophorus* potentiated their antifungal activity. These results could be related to an increase in production or diversification of active substances, reinforcing the possibility of a new type of chemical control. Thus, the co-cultivation approach is an interesting alternative for the discovery of new compounds and opens new perspectives for co-cultivations using other microorganisms potentially harmful to the ants’ nest.

Essential oils (EOs) are also considered efficient alternatives for the development of new agricultural products, since they show actions on different pathogens and can be safe for non-target organisms and the environment. In this way, *Myrcia lundiana* EO effectively inhibited the mycelium growth of *L. gongylophorus*. Isopulegol (**N21**) and citral (**N12**) were the major compounds identified for each EO chemotype and showed action against this symbiotic fungus. However, only **N12** was able to potentiate this inhibitory effect at a concentration three-fold lower than its respective chemotype (Table 3). The mechanism of action of **N12** is related to mitochondrial changes that modify the respiratory rate of fungal cells. In addition, 1-8-cineol had no fungistatic effect, although high concentrations of it were found in both EOs [67]. These results may be related to the chemical complexity of EOs, which contain a wide variety of compounds but generally only the major ones are considered bioactive. However, a loss of activity was observed when pure compounds were evaluated, suggesting that the use of the EO would be the best option due to the presence of a synergistic effect among its constituents.

On the other hand, dillapiole (**N16**) inhibited 95% of growth of *L. gongylophorus* at a concentration of 122 ppm (Table 3). This compound is among the major constituents of the *Piper holtonii* EO, which showed a fungicidal effect at 1000 ppm and, therefore, purification resulted in an eight-fold loss in activity [68]. All these data are important in proving the antifungal potential of EOs; however, no other work has reported this activity on *L. gongylophorus*, reinforcing the possibility of exploring other EOs as agents to control leaf-cutting ants.

The chemical composition of the extracts may vary according to biotic and abiotic factors; therefore, analyses of chemical identification and isolation are crucial in indicating bioactive substances. Medium-low polarity extracts of *C. fruticosa* have greater growth inhibition against *L. gongylophorus* than do methanolic extracts [69]. However, the fractionation of hexane and dichloromethane extracts reduced this effect on the symbiotic fungus, suggesting that loss of active compounds may occur during the separation process or that the antifungal potential is related to the synergistic effect between constituents of the extracts. Therefore, when starting a study for the discovery of compounds with biological activity, it is necessary that a biomonitored fractionation is performed in order to confirm whether the activity is related to only one constituent of the extract or whether there is a synergistic effect between them.

Fractionation of the ethanolic extract of *A. argentina* potentiated the inhibition of *L. gongylophorus* growth at a concentration of 50 µg/cm^2^. Refractionation allowed the isolation of argentilactone (**N9**), a lactone that showed a minimum inhibitory concentration value half that of the original extract [63]. Similarly, the performance of a guided bioassay led to the isolation of **N16**, which potentiated the antifungal activity of *P. holtonii* extract almost three-fold [68], as well as allowing the isolation of the fungicidal compound caryophyllene epoxide (**N11**) from the *Hymenaea courbaril* extract (Table 3) [70].

**Figure 2:**
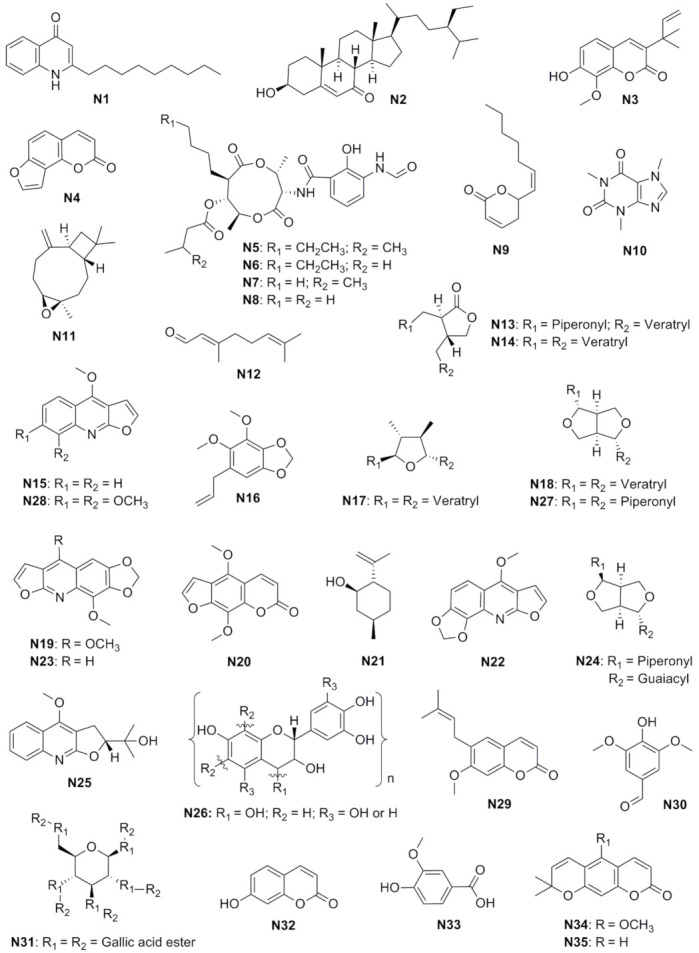
Chemical structure of natural compounds with potential antifungal effects against *Leucoagaricus gongylophorus*.

No difference in the percentage of growth inhibition of *L. gongylophorus* was observed between the extracts from the leaves and bark of *Raulinoa echinata*. However, the concentration of the hexane fraction that produced the same effect was reduced by half when compared to its respective crude extract. In sequence, the isolated compounds 2-*n*-nonyl-4-quinolone (**N1**), flindersiamine (**N19**), kokusagine (**N22**), maculine (**N23**), and skimmianine (**N28**) were even more effective in inhibiting fungal growth [17] (Table 3). All these compounds are examples of alkaloids, of which the furoquinolines are more active than the quinolones. The addition of a methoxy group to the furoquinoline alkaloids favored the antifungal activity compared to a carboxyl group (Figure 2). Caffeine (**N10**), an alkaloid found in a wide variety of species, also completely inhibited fungal growth, reinforcing the potential of this compound as a fungicidal agent [71].

Biological and chemical studies of *Pilocarpus grandiflorus* extract showed its inhibitory effect on *L. gongylophorus* growth, as well as allowing the identification of 18 substances. Among these, 3β-hydroxystigmast-5-en-7-one (**N2**), dictamine (**N15**), platydesmine (**N25**), syringaldehyde (**N30**), and vanillic acid (**N33**) also reproduced this biological effect. The last three compounds were effective at concentrations 20-fold lower than that of the extract (Table 3) [72].

A previous study had already shown that leaf-cutting ants have a selective consumption of leaves of three different species, with *Virola sebifera* being the last to be chosen [73]. This species contain lignans such as (2R,3R)-3-(3″,4″-dimethoxybenzyl)-2-(3′,4′-methylenedioxybenzyl)-butyrolactone (**N13**), (2R,3R)-2,3-di-(3′,4′-dimethoxybenzyl)-butyrolactone (**N14**), epigalgavrin (**N17**), eudesmin (**N18**), and sesamin (**N27**), and all of them inhibit the growth *L. gongylophorus*. Another antifungal lignan, philygenol (**N24**), was obtained from *Otoba parvifolia* [74]. Sesamin (**N27**) presented a remarkable antifungal activity at the lowest concentration compared to those of the other lignans (Table 3). Among the furofuran compounds, a reduction in fungal growth inhibition was observed when the piperonyl group was replaced by veratryl or guaiacyl rings, suggesting that the fungicidal effect is related to the presence of the first group. Interestingly, the opposite effect was observed for dibenzylbutyrolactone, that is, the activity increased when the piperonyl group was lost or replaced (Figure 2).

Coumarins form another class of secondary metabolites that have shown potent antifungal activity. Among the examples (Table 3), xanthyletin (**N35**) was the most effective in completely inhibiting the growth of *L. gongylophorus* at a concentration 2.5-fold lower than that of the others. Based on the structure–activity relationship, the addition of the methoxy group reduced the biological effect of xanthoxyletin (**N34**). On the other hand, this group favored the action of isopimpinellin (**N20**) when compared to angelicin (**N4**). Similarly, the methylation of the hydroxyl group at position 7 and the presence of a side chain at position 6 enhanced the antifungal activity of suberosin (**N29**) based on 7-hydroxy-3-(1′1′-dimethylallyl)-8-methoxycoumarin (**N3**) and umbelliferone (**N32**) (Figure 2) [75]. The presence of hydroxyl and/or ether groups at carbons 6 or 7 of the basic structure may favor the activity of these compounds [76].

In addition to the direct evaluation of microorganism growth, the quantification of enzymes essential for their growth can inform whether a compound has antifungal activity. *L. gongylophorus* produces a series of enzymes that act to protect itself and the leaf-cutting ants, such as polyphenol oxidase (PPO), an enzyme responsible for the detoxification of some secondary metabolites harmful to the ants’ nest [77,78]. In this sense, inhibition of this enzyme can leave the nest vulnerable and lead to the death of this symbiotic fungus. Quebracho tannin (**N26**) and tannic acid (**N31**) (Table 3) were evaluated as potent enzyme inhibitors, but only quebracho tannin was active [76,77], although both were able to reduce the fungal mass, suggesting that these tannins present different mechanisms of action. This difference can also be related to the structure of the compounds, since condensed tannins generally show greater antifungal effects than hydrolyzable ones (Figure 2) [77,78].

In the symbiotic behavior of Attine ants, the presence of microorganisms such as *Streptomyces* species guarantees protection to the nest due to the production of antimicrobial compounds. However, some of these compounds can also be harmful to the fungus garden [79]. For example, the antimycins A1–A4 (**N5**–**N8**) isolated from *Streptomyces* sp. had an inhibitory effect on both pathogenic fungi and *L. gongylophorus*. The mechanism of action of this class is related to the induction of apoptosis of fungal cells since they are inhibitors of the electron transfer of ubiquinol: cytochrome *C* reductase [79]. Thus, the complexity of symbiotic relationships is important for the survival of the leaf-cutting ant colonies; therefore, the lack of complete knowledge of how they work makes it difficult to control this pest. Thus, the antifungal activity of these lactones suggests that an internal imbalance in the nest would be crucial for its maintenance and opens up a new perspective for the discovery of active substances produced by the symbiotic microorganisms.Added to the antifungal activity, an insecticidal action would also be expected for a product to be effective in combatting leaf-cutting ants. Thus, among the extracts/compounds presented in the previous tables (Table 1, Table 2 and Table 3), *A. argentina, F. oolepis* [63], *C. fruticose* [69], *C. baccatum, C. frutescens* [65], and *Myrcia lundiana* and its major compounds citral and isopulegol [67] and caryophyllene epoxide [71] were also evaluated in the same works for their effects on leaf-cutting ants as potential multi-target control agents. This perspective also has advantages in reducing the emergence of resistant microorganisms compared to substances that show only one mode of action.

**Table 1:** Plant extracts and essential oils with potential antifungal effects against *Leucoagaricus gongylophorus * by in vitro assays.

Species	Extract	Part of Plant	Inhibitory Effect	Reference
*Achyrocline tomentosa*	Ethanol extract	Leaves	500 µg/spot–5 mm * (B)	[63]
*Ageratum conyzoides*	Hexane extract	Leaves	25 mg/mL–81% (C)	[64]
*Allium cepa*	-	Bulbs	IC_50_–1241.55 μg/mL (A)/2000 µg/mL–0.0 to 0.02 g ** (D)/2000 µg/mL–Fungistatic (E)	[19]
*Allium sativum*	-	Seed pods	IC_50_–358.36 μg/mL (A)/2000 µg/mL–0.0 g ** (D)/2000 µg/mL–Fungicidal (E)	[19]
*Aristolochia argentina*	Ethanol extract	-	500 µg/spot–15 mm * (B)/MIC–1.95 µg/mL (F)	[63]
*Baccharis linearifolia*	Ethanol extract	-	500 µg/spot–5 mm * (B)	[63]
*Baccharis coridifolia*	Ethanol extract	-	500 µg/spot–5 mm * (B)	[63]
*Baccharis flabellata*	Ethanol extract	-	500 µg/spot–10 mm * (B)	[63]
*Capsicum baccatum*	Ethanol extract	Leaves	0.1% *w*/*v*–40% (A)	[65]
*Capsicum frutescens*	Ethanol extract	Leaves	0.1% *w*/*v*–20% (A)	[65]
*Cipadessa fruticosa*	Dichloromethane extract	Fruits	1000 mg/mL–80% (A)	[69]
	Hexane extract	Branches	1000 mg/mL–80% (A)	
	Dichloromethane extract	Branches	1000 mg/mL–40% (A)	
	Hexane extract	Leaves	1000 mg/mL–20% (A)	
	Dichloromethane extract	Leaves	1000 mg/mL–20% (A)	
*Coriandrum sativum*	Hexane extract	Leaves	100 mg/mL–100% (A)	[64]
*Dalea elegans*	Ethanol extract	-	500 µg/spot–10 mm * (B)	[63]
*Flourensia oolepis*	Ethanol extract	-	500 µg/spot–5 mm * (B)/MIC–7.8 µg/mL (F)	[63]
*Grindelia pulchella*	Ethanol extract	-	500 µg/spot–5 mm * (B)	[63]
*Lycopersicon esculentum*	-	Green fruits	IC_50−_2262.29 μg/mL (A)/2000 µg/mL–0.02 to 0.05 g ** (D)/2000 µg/mL–Fungistatic (E)	[19]
*Manihot esculenta*	-	Leaves	IC_50−_553.32 μg/mL (A)/2000 µg/mL–0.0 g ** (D)/2000 µg/mL–Fungicidal (E)	[19]
*Mentha piperita*	Hexane extract	Leaves	25 mg/mL–96% (C)	[64]
*Myrcia lundiana*	Essential oil (Citral chemotype)	Leaves	IC_50−_104.8 μL/L ^#^ (A)/IC_50–_217.9 µL/L ^##^ (A)	[67]
	Essential oil (Isopulegol chemotype)	Leaves	IC_50−_145.1 μL/L ^#^ (A)/IC_50–_238.1 µL/L ^##^ (A)	
*Pilocarpus grandiflorus*	Dichloromethane extract	Stems	1000 µg/mL–10% (A)	[72]
*Piper holtonii*	Ethanol extract	Leaves	IC_50−_102 ppm (A)	[68]
	Essential oil	Leaves	1000 ppm–100% (A)	
*Pterocaulon alopecuroides*	Ethanol extract	-	500 µg/spot–15 mm * (B)/MIC–7.8 µg/mL (F)	[63]
*Raulinoa echinata*	Methanol extract	Stems	1000 μg/mL–80% (A)	[17]
	Methanol extract	Leaves	1000 μg/mL–80% (A)	
	Methanol extract (hexane fraction)	Leaves	500 μg/mL–80% (A)	
*Senna alata*	-	Leaves	IC_50−_251.51 μg/mL (A)/500 µg/mL–0.0 g ** (D)/500 µg/mL–Fungicidal (E)	[19]
*Sesamum indicum*	Chloroform extract	Leaves	60 mg/mL–60% (A)	[8]
	Methanol extract	Leaves	60 mg/mL–60% (A)	
	Methanol + Chloroform extract	Leaves	60 + 60 mg/mL–>80% (A)	
	Chloroform extract	Leaves (30 days old)	60 mg/mL–60% (A)	
	Chloroform extract	Leaves (60 days old)	60 mg/mL–60% (A)	
	Chloroform extract	Green leaves (90 days old)	60 mg/mL–40% (A)	
	Chloroform extract	Yellow leaves (90 days old)	60 mg/mL–60% (A)	
	Chloroform extract	Green fruit	30 mg/mL–40% (A)	
	Chloroform extract	Ripe fruit	30 mg/mL–60% (A)	
	Chloroform extract	Green seed	30 mg/mL–60% (A)	
	Chloroform extract	Ripe seed	30 mg/mL–60% (A)	
*Trichocline reptans*	Ethanol extract	-	500 µg/spot–5 mm * (B)	[63]
*Zanthoxylum coco*	Ethanol extract	-	500 µg/spot–10 mm * (B)	[63]

Antifungal assay: (A) agar assay mycelium growth; (B) bioautography assay; (C) fungal biomass reduction by agar assay; (D) fungal biomass reduction by broth assay; (E) MTT assay; (F) broth microdilution assay. MIC: minimum inhibitory concentration. IC_50_: concentration that inhibits 50% of the fungal growth. * Mean inhibition area. ** Fungus weight. ^#^ Fumigation assay. ^##^ Contact assay.

**Table 2:** Microorganisms extracts with potential antifungal effects against *Leucoagaricus gongylophorus* by in vitro assays.

Microorganism	Strain	Extract	Inhibitory Effect (Antifungal Assay)	Reference
*Escovopsioides nivea*	LESF596 + Absence of *L. gongylophorus*	Crude extract	3–5 cm^2^ * (A)	[66]
	LESF596 + Presence of *L. gongylophorus*	Crude extract	3–5 cm^2^ * (A)	
	LESF599 + Absence of *L. gongylophorus*	Crude extract	4–5 cm^2^ * (A)	
	LESF599 + Presence of *L. gongylophorus*	Crude extract	4–5 cm^2^ * (A)	
*Escovopsis* sp.	LESF017 + Absence of *L. gongylophorus*	Crude extract	3–5 cm^2^ * (A)	[66]
	LESF017 + Presence of *L. gongylophorus*	Crude extract	2–3 cm^2^ * (A)	
	LESF019 + Absence of *L. gongylophorus*	Crude extract	4–5 cm^2^ * (A)	
	LESF019 + Presence of *L. gongylophorus*	Crude extract	3–4 cm^2^ * (A)	
	LESF021 + Absence of *L. gongylophorus*	Crude extract	2–4 cm^2^ * (A)	
	LESF021 + Presence of *L. gongylophorus*	Crude extract	1–3 cm^2^ * (A)	
	LESF023 + Absence of *L. gongylophorus*	Crude extract	4–6 cm^2^ * (A)	
	LESF023 + Presence of *L. gongylophorus*	Crude extract	3–5 cm^2^ * (A)	
	LESF033 + Absence of *L. gongylophorus*	Crude extract	1–3 cm^2^ * (A)	
	LESF033 + Presence of *L. gongylophorus*	Crude extract	1–2 cm^2^ * (A)	
	LESF039 + Absence of *L. gongylophorus*	Crude extract	3–5 cm^2^ * (A)	
	LESF039 + Presence of *L. gongylophorus*	Crude extract	3–4 cm^2^ * (A)	
	LESF040 + Absence of *L. gongylophorus*	Crude extract	4–6 cm^2^ * (A)	
	LESF040 + Presence of *L. gongylophorus*	Crude extract	3–5 cm^2^ * (A)	
	LESF041 + Absence of *L. gongylophorus*	Crude extract	3–5 cm^2^ * (A)	
	LESF041 + Presence of *L. gongylophorus*	Crude extract	5–6 cm^2^ * (A)	
	LESF043 + Absence of *L. gongylophorus*	Crude extract	4–5 cm^2^ * (A)	
	LESF043 + Presence of *L. gongylophorus*	Crude extract	3–4 cm^2^ * (A)	
	LESF045 + Absence of *L. gongylophorus*	Crude extract	1–3 cm^2^ * (A)	
	LESF045 + Presence of *L. gongylophorus*	Crude extract	3–5 cm^2^ * (A)	

Antifungal assay: (A) agar assay mycelium growth. * Fungal growth area.

**Table 3:** Isolated compounds with potential antifungal effects against *Leucoagaricus gongylophorus* by in vitro assays.

Compound	Class	Species (Part)	Characterization Method	Inhibitory Effect	Reference
2-*n*-Nonyl-4-quinolone (**N1**)	Alkaloid	*Raulinoa echinata* (leaves)	^1^H NMR, ^13^C NMR, and EIMS	100 μg/mL–50% (A)	[17]
3β-Hydroxystigmast-5-en-7-one (**N2**)	Steroid	*Pilocarpus grandiflorus* (stems)	-	60 µg/mL–20% (A)	[72]
7-Hydroxy-3-(1′1′-dimethylallyl)-8-methoxycoumarin (**N3**)	Coumarin	*Pilocarpus riedelianus* (stems)	^1^H NMR, ^13^C NMR, MS and IR	75 µg/mL–80% (A)	[75]
Angelicin (**N4**)	Coumarin	*Citrus limonia* (roots)	^1^H NMR, ^13^C NMR, MS and IR	72 µg/mL–40% (A)	[75]
Antimycin A1 (**N5**)	Macrolide	*Streptomyces* sp.	LC-ESI-MS	5.8 nmol–+ * (B)	[79]
Antimycin A2 (**N6**)	Macrolide	*Streptomyces* sp.	LC-ESI-MS	5.8 nmol–+ * (B)	[79]
Antimycin A3 (**N7**)	Macrolide	*Streptomyces* sp.	LC-ESI-MS	5.8 nmol–+ * (B)	[79]
Antimycin A4 (**N8**)	Macrolide	*Streptomyces* sp.	LC-ESI-MS	5.8 nmol–+ * (B)	[79]
Argentilactone (**N9**)	Fatty acid lactone derivative	*Aristolochia argentina* (aerial parts)	^1^H NMR, ^13^C NMR, and GC-MS	MIC–0.90 µg/mL (C)	[63]
Caffeine (**N10**)	Alkaloid	-	-	0.50% *w*/*v*–100% (A)	[71]
Caryophyllene epoxide (**N11**)	Terpene	*Hymenaea courbaril* (leaves)	GC-MS and ^13^C NMR	3 mg/mL–100% (A)	[72]
Citral (**N12**)	Terpene	*Myrcia lundiana* (leaves)	GC-MS and GC-FID	IC_50−_31.7 μL/L ^#^ (A)/IC_50−_289.9 µL/L ^##^ (A)	[67]
(2R,3R)-2,3-Di-(3′,4′-dimethoxybenzyl)-butyrolactone (**N13**)	Lignan	*Virola sebifera* (leaves)	-	200 μg/mL–20% (A)	[74]
(2R,3R)-3-(3″,4″-Dimethoxybenzyl)-2-(3′,4′-methylenedioxybenzyl)-butyrolactone (**N14**)	Lignan	*Virola sebifera* (leaves)	-	210 μg/mL–60% (A)	[74]
Dictamine (**N15**)	Alkaloid	*Pilocarpus grandiflorus* (stems)	-	40 µg/mL–40% (A)	[72]
Dillapiole (**N16**)	Phenylpropanoid	*Piper holtonii* (leaves)	GC-MS, ^1^H NMR, ^13^C NMR, and HMBC	IC_50−_38 ppm (A)	[68]
Epigalgavrin (**N17**)	Lignan	*Virola* sp. (leaves)	-	200 μg/mL–>80% (A)	[74]
Eudesmin (**N18**)	Lignan	*Virola sebifera* (leaves)	-	160 μg/mL–40% (A)	[74]
Flindersiamine (**N19**)	Alkaloid	*Raulinoa echinata* (stems)	^1^H NMR, ^13^C NMR, HMBC, and X-ray	100 μg/mL–50% (A)	[17]
Isopimpinellin (**N20**)	Coumarin	*Adiscanthus fusciflorus* (roots)	^1^H NMR, ^13^C NMR, MS, and IR	80 µg/mL–100% (A)	[75]
Isopulegol (**N21**)	Terpene	*Myrcia lundiana* (leaves)	GC-MS and GC-FID	IC_50−_150.1 μL/L ^#^ (A)/IC_50−_696.8 µL/L ^##^ (A)	[67]
Kokusagine (**N22**)	Alkaloid	*Raulinoa echinata* (stems)	^1^H NMR and ^13^C NMR	100 μg/mL–100% (A)	[17]
Maculine (**N23**)	Alkaloid	*Raulinoa echinata* (stems)	^1^H NMR and ^13^C NMR	100 μg/mL–50% (A)	[17]
Philygenol (**N24**)	Lignan	*Otoba parvifolia* (fruits)	-	200 μg/mL–20% (A)	[74]
Platydesmine (**N25**)	Alkaloid	*Pilocarpus grandiflorus* (stems)	-	50 µg/mL–80% (A)	[72]
Quebracho tannin (**N26**)	Tannin	-	-	0.25% *w*/*v*–-0.02 ^&^ (D)/0.25% *w*/*v*–0.0 to 0.5 mg ^&&^ (E)	[78]
Sesamin (**N27**)	Lignan	*Virola sebifera* (leaves)	-	70 μg/mL–>80% (A)	[74]
Skimmianine (**N28**)	Alkaloid	*Raulinoa echinata* (stems)	^1^H NMR and ^13^C NMR	100 μg/mL–80% (A)	[17]
Suberosin (**N29**)	Coumarin	*Citrus limonia* (roots)	^1^H NMR, ^13^C NMR, MS, and IR	64 µg/mL–100% (A)	[75]
Syringaldehyde (**N30**)	Benzaldehyde	*Pilocarpus grandiflorus* (stems)	-	50 µg/mL–80% (A)	[72]
Tannic acid (**N31**)	Tannin	-	-	0.25% *w*/*v*–0.08 ^&^ (D)/0.025% *w*/*v*–0.5 to 1.0 mg ^&&^ (E)	[77]
Umbelliferone (**N32**)	Coumarin	*Picramnia teapensis* (bark)	^1^H NMR, ^13^C NMR, MS, and IR	65 µg/mL–60% (A)	[75]
Vanillic acid (**N33**)	Phenolic acid	*Pilocarpus grandiflorus* (stems)	-	50 µg/mL–80% (A)	[75]
Xanthoxyletin (**N34**)	Coumarin	*Citrus limonia* (roots)	^1^H NMR, ^13^C NMR, MS, and IR	70 µg/mL–100% (A)	[75]
Xanthyletin (**N35**)	Coumarin	*Pilocarpus riedelianus* (stems)	^1^H NMR, ^13^C NMR, MS, and IR	25 µg/mL–100% (A)	[75]

Antifungal assay: (A) agar assay mycelium growth; (B) agar diffusion assay; (C) broth microdilution assay; (D) polyphenol oxidase activity; (E) fungal biomass reduction by broth assay. MIC: Minimum inhibitory concentration. IC_50_: concentration that inhibits 50% of the fungal growth. * Presence of antifungal effect (percentage inhibition not reported by the author). ^#^ Fumigation assay. ^##^ Contact assay. ^&^ Relative optical density. ^&&^ Fungal biomass.

#### 2.1.2. Synthetic Compounds

Although the chemical variety of natural products is extensive, synthetic compounds have advantages related to purity as well as the ease of large-scale production [80]. Various synthetic products showed toxic effects against *L. gongylophorus* (Table 4), including fatty acids with 6–12 carbons (**S12**–**S18**) [61]. Although no differences in activity were observed among them, the inhibition of fungal growth can vary according to the number of carbon atoms: the longer the chain, the greater the biological effect. This observation can be proven from the synthesis of piperonyl compounds, since an increase in the length of the side chain from two to eight carbons (**S1**–**S4**) reduced the concentration effective against the symbiotic fungus almost 10-fold. However, this relationship is not continuous, as a reduction in antifungal activity was observed for side chains with more than 10 carbons (**S5**) [81].

**Figure 3:**
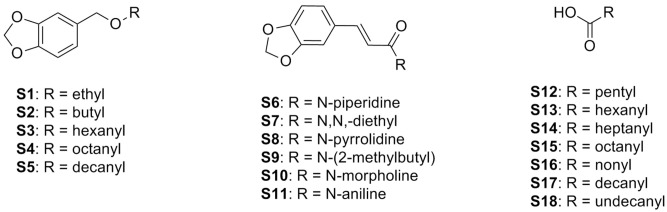
Chemical structure of synthetic compounds with potential antifungal effect against *Leucoagaricus gongylophorus*.

In addition to the carbon chain, the presence of the piperonyl group could be responsible for the antifungal effect of lignans [74], corroborating the results of synthetic piperonyl compounds. In this way, 1-(3,4-methylenedioxybenzyloxy)octane (**S4**), containing the piperonyl group and an 8-carbon side chain (Figure 3), showed the best antifungal result among the synthetic compounds at a concentration 4.5-fold lower than that of the natural lignan sesamin (**N27**) (Table 4). Thus, structural modification can be an important strategy in enhancing the biological effect and improving the physical-chemical characteristics, such as the solubility of natural compounds with antifungal activity against *L. gongylophorus*.

Some amides and lignans protect plants against attacks by insects and other pathogens. Piperamides have been synthesized to enhance the biological effect of both groups [82]. This improvement can be confirmed by the antifungal activity of the compounds *N*-piperidine-3-(30,40-methylenedioxyphenyl)-2-(E)-propenamide (**S6**) and *N,N*-diethyl-3-(3,4-methylenedioxyphenyl)-2-(E)-propenamide (**S7**), which inhibit the growth of *L. gongylophorus* by 100% at 50 µg/mL. This result is even more promising when compared to those of the natural lignans shown in Table 3, in which only argentilactone (**N9**) showed a lower effective concentration (0.90 µg/mL). In addition, **S6** and **S7** showed greater antifungal potential than synthetic piperonyl compounds **S1**, **S2**, **S3**, and **S5**, suggesting that the addition of the amide group improves their biological effect (Figure 3).

Filamentous fungi can be found in different environments; their ability to adapt is related to the production of enzymes such as those belonging to the cytochrome P450 superfamily, responsible for the degradation of complex substances [83]. The presence of a methylenedioxyphenyl group may be responsible for the antifungal activity presented by synthetic piperonyl and piperamide compounds, since this group is a cytochrome P450 inhibitor [82]. In this way, the processes of xenobiotic biotransformation and detoxification reactions catalyzed by the cytochrome P450 would be downregulated, increasing the susceptibility of the microorganisms to the compound.

All compounds described in Table 4 were also evaluated for their insecticidal potential, aiming for a dual-action strategy for chemical products that would be advantageous in controlling leaf-cutting ants [62,81,82]. Forest companies experience an average cost for chemical insect pest control (USD 29.50/ha/year) about 64-fold higher than their spending on biological control (USD 0.46/ha/year), possibly related to the high price of chemicals and the technologies required for their application [59]. In this sense, in addition to achieving the satisfactory control of leaf-cutting ants, a cost–benefit assessment for the products is also necessary.

**Figure 4:**
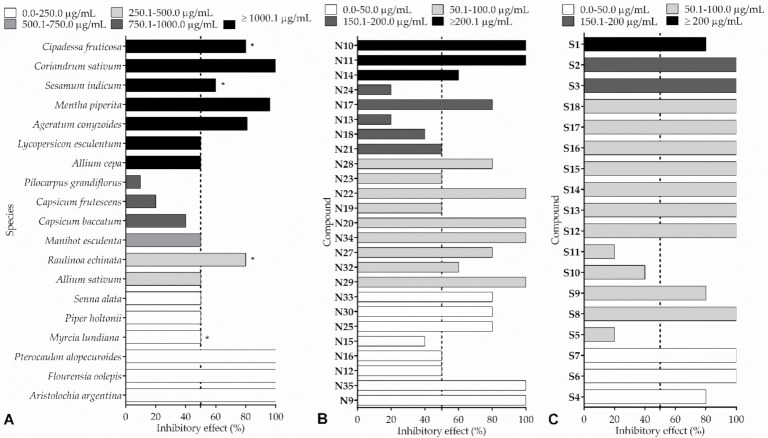
Relationship of the percentage of inhibitory effect on *Leucoagaricus gongylophorus* growth according to the concentration of extracts (**A**), isolated natural compounds (**B**), and synthetic compounds (**C**). Graph obtained only from results expressed as a percentage inhibitory effect. Other data expressions were excluded due to the difficulty of comparison, but can be found in Table 1, Table 2, Table 3 and Table 4. * The most active extract was selected for species with more than one type of extract.

Figure 4 summarizes the relation between the percentage of *L. gongylophorus* growth reduction according to the concentration of the extracts or compounds. This approach is not ideal, since differences in methodologies (inoculum concentration, incubation time, type of culture medium, etc.) can interfere with the results. In general, the species *A. argentina*, *F. oolepis,* and *P. alopecuroides* showed promise as candidates in the search for bioactive substances, as they totally inhibit the fungal growth at the lowest concentrations (Figure 4A). Similarly, compounds **N9** and **N35** can also be highlighted for their fungicidal activity, and, although compounds **N25**, **N30,** and **N33** showed a weaker action compared to the former compounds, a small increase in concentration may provide a similar antifungal activity (Figure 4B). Finally, the synthesized substances **S4**, **S6,** and **S7** are also indicated as novel control agents (Figure 4C). However, it is difficult to select the species or compounds with the greatest antifungal potential when different methodologies are applied; therefore, further studies should be carried out to evaluate these data. 

**Table 4:** Synthetic compounds with potential antifungal effects against *Leucoagaricus gongylophorus* by in vitro assays.

Compound	Class	Characterization Method	Inhibitory Effect (Antifungal Assay)	Reference
1-(3,4-Methylenedioxybenzyloxy)ethane (**S1**)	Piperonyl-alkane	^1^H NMR, ^13^C NMR, and EIMS	330 μg/mL–80% (A)	[81]
1-(3,4-Methylenedioxybenzyloxy)butane (**S2**)	Piperonyl-alkane	^1^H NMR, ^13^C NMR, and EIMS	170 μg/mL–100% (A)	
1-(3,4-Methylenedioxybenzyloxy)hexane (**S3**)	Piperonyl-alkane	^1^H NMR, ^13^C NMR, and EIMS	160 μg/mL–100% (A)	
1-(3,4-Methylenedioxybenzyloxy)octane (**S4**)	Piperonyl-alkane	^1^H NMR, ^13^C NMR, and EIMS	15 μg/mL–80% (A)	
1-(3,4-Methylenedioxybenzyloxy)decane (**S5**)	Piperonyl-alkane	^1^H NMR, ^13^C NMR, and EIMS	100 μg/mL–20% (A)	
N-Piperidine-3-(3,4-methylenedioxyphenyl)-2-(E)-propenamide (**S6**)	Piperonyl-amide	^1^H NMR, ^13^C NMR, MS, IR, and TLC	50 µg/mL–100% (A)	[82]
N,N-Diethyl-3-(3,4-methylenedioxyphenyl)-2-(E)-propenamide (**S7**)	Piperonyl-amide	^1^H NMR, ^13^C NMR, MS, IR, and TLC	50 µg/mL–100% (A)	
N-Pyrrolidine-3-(3,4-methylenedioxyphenyl)-2-(E)-propenamide (**S8**)	Piperonyl-amide	^1^H NMR, ^13^C NMR, MS, IR, and TLC	100 µg/mL–100% (A)	
N-(2-Methylbutyl)-3-(3,4-methylenedioxyphenyl)-2-(E)-propenamide (**S9**)	Piperonyl-amide	^1^H NMR, ^13^C NMR, MS, IR, and TLC	100 µg/mL–80% (A)	
N-Morpholine-3-(3,4-methylenedioxyphenyl)-2-(E)-propenamide (**S10**)	Piperonyl-amide	^1^H NMR, ^13^C NMR, MS, IR, and TLC	100 µg/mL–40% (A)	
N-Aniline-3-(3,4-methylenedioxyphenyl)-2-(E)-propenamide (**S11**)	Piperonyl-amide	^1^H NMR, ^13^C NMR, MS, IR, and TLC	100 µg/mL–20% (A)	
Hexanoic acid (**S12**)	Saturated fatty acid	-	100 µg/mL–100% (A)	[62]
Heptanoic acid (**S13**)	Saturated fatty acid	-	100 µg/mL–100% (A)	
Octanoic acid (**S14**)	Saturated fatty acid	-	100 µg/mL–100% (A)	
Nonanoic acid (**S15**)	Saturated fatty acid	-	100 µg/mL–100% (A)	
Decanoic acid (**S16**)	Saturated fatty acids	-	100 µg/mL–100% (A)	
Undecanoic acid (**S17**)	Saturated fatty acid	-	100 µg/mL–100% (A)	
Lauric acid (**S18**)	Saturated fatty acid	-	100 µg/mL–100% (A)	

Antifungal assay: (A) agar assay mycelium growth.

### 2.2. Biological Control Using Microorganisms

As previously discussed, leaf-cutting ants live in symbiosis with other microorganisms that assist in their feeding and protection. However, drastic changes in the environment in which they live can affect the balance of the nest, favoring the development of other invading organisms [84]. Among them, the species of the genera *Escovopsis* [66,84,85] and *Trichoderma* [84,86,87] can be highlighted as the main threats (Table 5). *Escovopsioides nivea* is also effective in reducing fungal mycelium development [66], and lower values of inhibition were demonstrated for *Acremonium kiliense* [84] and *Gliocladium* sp. [87] species. However, *Syncephalastrum* sp. showed a harmful effect on sub-colonies regardless of the occurrence of the disturbance to the ants’ nest, suggesting that it may be susceptible to attack by pathogenic microorganisms even under normal conditions (healthy sub-colonies) [88].

When the nest is under attack by invading microorganisms, there is a reduction in biomass in the fungus garden since they consume the nutritional content of *L. gongylophorus* [84]. Inhibitory effect on the fungus garden shown by *A. kiliense* is related to this nutritional competition [84]. The degradation of the symbiotic fungus produces substances that can also be used as a food source for the species of *Escovopsis* and *Trichoderma*, indicating a necrotrophic relationship between them [84]. A combination of mechanisms of action can also be found for the same microorganism, such as *Gliocladium*, which both inhibits development and competes for nutrients [87]. Other mechanisms of action, such as the production of substances with antibiotic potential or that degrade the cell wall, may also be involved in the antifungal control [86].

Different microorganism strains, fungi, or bacteria may have different mechanisms on *L.* gongylophorus since the genetic homogeneity of this mutualistic fungus restricts its capacity for defense against different organisms [85]. This can be observed in the increase in the percentage of mycelium growth inhibition from 43 to 78% after the attack by *Escovopsis* strains [66,84,85]. *Trichoderma* species also showed large divergences (1–75%) in inhibition of the growth of *L. gongylophorus* (Table 5) [84,86,87].

Although *L. gongylophorus* is susceptible to other microorganisms, their direct application in the field would not be viable since this effect would probably be lost over time. This is because leaf-cutting ants exhibit highly specialized hygiene behavior that allows the creation of barriers to invasion by other microorganisms as well as being able to eliminate them [66,84,85,88]. However, microorganisms have primary and secondary metabolisms similar to those of plants; therefore, they can be considered a rich source of active substances for chemical control. As examples, the lactones antimycins A1–A4 (**N5**–**N8**), obtained from *Streptomyces* sp. [79], and extracts of *E. nivea* and *Escovopsis* [66] suppress the growth of *L. gongylophorus*.

Conventional biological control methods using the direct application of microorganisms in the environment do not seem to be the ideal way to combat this crop pest due to the hygienic behavior of the leaf-cutting ants. Thus, metabolomics research could be an alternative in the search for novel compounds active against *L. gongylophorus*. On the other hand, pathogenic fungi have already shown insecticidal action better than that of chemical controls, suggesting that the application of a biological control on leaf-cutting ants may be possible and applicable [89]. Ideally, pathogenic fungi would act faster than the ability of leaf-cutting ants to eliminate them, and that would have an action specific to these insects; so, microorganisms that avoid any environmental imbalance would be preferable for biological control. Little is known about how these organisms interact in the fungus garden, and further studies are needed into the mechanism of the antifungal action.

**Table 5:** Microorganisms with potential antifungal effects against *Leucoagaricus gongylophorus * by in vitro assays.

Organism	Strain	Inhibitory Effect (Antifungal Assay)	Reference
*Acremonium kiliense*	C1	26% (A)	[84]
*Escovopsioides nivea*	LESF596	56% (A)	[67]
	LESF599	45% (A)	
*Escovopsis* sp.	AP090209–01	+ * (A)	[85]
	AP090225-01	+ * (A/B/C)	
	AP090731-01	+ * (A/C)	
	AP100526-01	+ * (A/B/C)	
	DE090731-01	+ * (A/C)	
	LD100306-01	+ * (B/C)	
	RM090730-01	+ * (B)	
	RM090730-02	+ * (A/B/C)	
	LESF017	78% (A)	[67]
	LESF043	70% (A)	
	LESF041	68% (A)	
	LESF039	65% (A)	
	LESF019	64% (A)	
	LESF021	62% (A)	
	LESF045	61% (A)	
	LESF033	59% (A)	
	LESF040	58% (A)	
	LESF023	56% (A)	
*Escovopsis weberi*	CBS 810.71	68% (A)	[85]
	A088	67% (A)	
	A086	43% (A)	
*Gliocladium* sp.	G-56	9% (A)	[87]
	G-55	10% (A)	
*Syncephalastrum* sp.	LESF130	8.71 ± 1.85 mm² ** (A)	[88]
	LESF125	8.22 ± 1.64 mm² ** (A)	
	LESF127	8.80 ± 1.60 mm² ** (A)	
*Trichoderma koningii*	T-83	1% (A)	[87]
*Trichoderma harzianum*	T-21	22% (A)	[87]
	T-86	30% (A)	
*Trichoderma koningiopsis*	HEP4	67.37% (A)	[86]
	HEP12	69.78% (A)	
	HEP20	58.03% (A)	
*Trichoderma lignorum*	T-28	11% (A)	[87]
	T-19	32% (A)	
	T-26	53% (A)	
	T-20	6% (A)	
	T-30	28% (A)	
*Trichoderma* sp.	T-22	4% (A)	[87]
	T-27	4% (A)	
	T-24	9% (A)	
	T-29	42% (A)	
	T-109	47% (A)	
	T-110	9% (A)	
	T-71	19% (A)	
*Trichoderma viridae*	T-25	21% (A)	[89]
	T-23	33% (A)	

Antifungal assay: (A) agar assay mycelium growth; (B) fungus garden inhibitory bioassay; (C) sub-colony bioassay. * Presence of antifungal effect (percentage inhibition not reported by the author). ** Fungal mycelium growth area informed by the author (percentage calculated: LESF130–48%; LESF125–51%; LESF127–48%).

## 3. Future Perspectives

### 3.1. Standardization of Antifungal Assay

**Figure 5:**
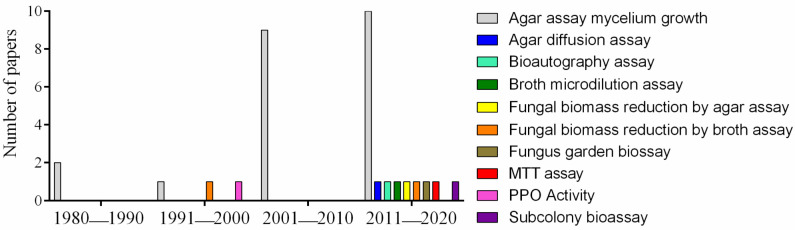
Different types of antifungal methodologies performed on *Leucoagaricus gongylophorus* according to each decade.

As shown in Figure 5, different methods have been used to evaluate the effect against the growth of *L. gongylophorus*. Initially, the assays were based only on the observation of mycelium growth in agar. Over the years, methodologies have been diversified and improved to obtain more reliable results. The comparison of results between various works is a crucial point in the selection of species and compounds with the greatest potential to suppress the fungus garden. However, the difference in methodologies makes this analysis difficult and allows only the indication of the best products within the same work.

Although there are differences between the methodologies developed so far, most of them represent in vitro analyses. However, Wallace et al. [85] used a sub-colony bioassay to verify the action of *Escovopsis* against *L. gongylophorus*, with this being the method that is closest to the in vivo system. This approach allows the maintenance of the interactions between the organisms that make up the nest, with these being the ants, the fungus garden, and the associated microorganisms, as well as a food, which mimics that occurring in the environment. The ability to reproduce the data in vitro could also be verified, since five of the six strains that showed the best results in the agar growth assay were also the most virulent microorganisms. Therefore, it is important to note that, initially, in vitro analyses are preferred due to the ease and reproducibility of obtaining results from the screening of active substances. However, the standardization of a quantitative method for the selection of promising compounds and the development of more reliable methodologies for use under field conditions are also necessary to prove these data.

The National Committee for Clinical Laboratory Standards (NCCLS) recommends a reference method for broth dilution antifungal susceptibility testing of filamentous fungi (M38-A) [90], which could be applied to *L. gongylophorus* as it is an example of this class of microorganism. However, so far, no work has been based on this standard; therefore, new studies following this method to confirm its effectiveness in the evaluation of active substance against this symbiotic fungus, as well as to obtain a standardization of the results that can be reproduced and analyzed from anywhere in the world, would be highly recommended.

### 3.2. Evaluation of the Antifungal Mechanism of Action

Specific targets within the fungus organism are the goal of many applications of compounds with fungicidal properties. There are five different classes of antifungal agents (polyenes, azoles, allylamines, pyrimidines, and echinocandins); the action of four of them is related to the inhibition of the synthesis of ergosterol and cell wall glucans. Only pyrimidines act by inhibiting DNA and RNA biosynthesis through action on the pyrimidine metabolism [80].

EOs are important sources of natural products that act on the fungus membrane. For example, the EOs of *Aniba canelilla* and *A. parviflora* showed fungicidal activities against the pathogenic fungi of the plants *Alternaria alternata*, *Aspergillus flavus*, *A. niger*, *Colletotrichum gloeosporioides*, *C. guaranicola, C. musae*, *Fusarium oxysporum,* and *F. solani* [91]. Studies on the mechanism of action based on cell component leakage assay showed that *Aniba* EO can induce the release of nucleic acids and proteins by damaging the cell membrane integrity, leading to a lethal effect on the pathogens.

As examples of different antifungal agents, there are inhibitors of the mitochondrial function [92]. Boscalid, a pyridinecarboxamide, is a succinate dehydrogenase (SDH) inhibitor fungicide and is highly effective in controlling *A. alternata*, *Botrytis cinerea*, *Corynespora cassiicola, Penicillium digitatum,* and *Sclerotinia sclerotiorum* [93,94,95,96,97]. The enzyme SDH catalyzes the oxidation of succinate to fumarate in the mitochondrial matrix. This reactive oxygen species (ROS) plays a complex role in regulating the growth, development, and virulence of fungal pathogens and is produced as a bioproduct of aerobic respiration in fungi; thus, blockage of the respiratory chain by boscalid can affect ROS production [97].

The antifungal mechanism of action of **N12** is also related to its negative effect on the mitochondria [67]. Alteration in the fungal cell respiratory chain has also been indicated as the mechanism of action of **N5**–**N8** since they act by inhibiting electron transfer of ubiquinol: cytochrome *C* reductase [70]. Although this structure may be the target of action responsible for the inhibition of *L. gongylophorus* growth, no assay specifically on this microorganism has been performed.

In view of this limitation, the discovery of drugs with action targets different from those of commercially available products is necessary. Ideally, compounds with action on fungi-specific pathways are important sources of new agents, as they would show a reduction in toxicity for farmers as well as for crops [98].

The high degree of organization and hygiene of the leaf-cutting ant allows the organisms involved in the maintenance of the nest to be effective in eliminating toxic substances before their destruction takes place. Therefore, another promising alternative would be the use of products that act by inhibiting key enzymes crucial in the metabolization of these harmful substances, for example, the inhibition of cytochrome P450, which is responsible for xenobiotic biotransformation and detoxification reactions by compounds containing a methylenedioxyphenyl group [82]. Similarly, the inhibition of polyphenol oxidase, another detoxifying enzyme, has also resulted in an inhibitory effect on the growth of *L. gongylophorus* [78].

Knowledge available on fungi-specific pathways is limited; therefore, further studies should be conducted for the effective development of drugs for this purpose. In addition, there is a large gap in the knowledge of the modes of action of compounds used to combat phytopathogens since the greatest interest is still related to human pathogens [80,99]. Thus, it is important to highlight the need for mechanistic studies using microorganisms that damage agricultural production as target organisms, and the aforementioned pathways can be used as a source for analysis.

### 3.3. Product Development

As discussed throughout the manuscript, several research items proved that there is a great diversity of compounds and microorganisms capable of controlling the mutualist fungus of the leaf-cutting ants; however, although the laboratory results of these research items are promising, control agents against *L. gongylophorus* have hardly been evaluated in the field. The practical use of natural products in the field would be unviable due to the high chemical instability, as well as the low water solubility that would diminish their efficiency [67]. Development of delivery systems has demonstrated better results for the active ingredient, such as protection against degradation, controlled release, increased bioavailability, reduced damage to non-target organisms and the environment, and less development of resistant organisms [100,101,102]. In view of this reality, the development of or search for carriers such as the insecticide baits has emerged as a new approach in order to enable and enhance their biological use as eco-friendly agents for crop protection.

Apparently, the best way to deliver the compounds or microorganisms that control *L. gongylophorus* is in the form of baits, since the leaf-cutting ants themselves would be in charge of transporting them into the nest by foraging, serving as a substrate for the garden of mushrooms. In this regard, the carrier must meet requirements similar to those used in the production of insecticide baits such as dehydrated citrus pulp that is capable of attracting ants over long distances [21,23] because the effectiveness of the bait depends on the attractiveness of the substrate used, resulting in low or high carrying rates [23]. In addition, control agents against *L. gongylophorus* must be chemically and physically stable when exposed to adverse conditions, have a delayed toxic action, and present low bioaccumulation in the environment, as well as not repelling or not being identified as hazardous agents by leaf-cutting ants, in order to allow high transport rates and low return rates of the bait [20]. Since they are eusocial insects that efficiently divide labor within the colony, leaf-cutting ants have the ability to clean the nest, including the fungus garden, and secrete substances to protect other ants and the fungus against harmful microorganisms [103].

Nanoemulsions loaded with neem oil show a greater inhibitory effect on *A. flavus* and *Penicillium citrinum* than the unformulated form, as well as potentiating the germination of contaminated soybean seeds [104]. In the same way, nano-encapsulated EOs from *Majorana hortensis*, *M. piperita*, *Mentha spicata, Ocimum basilicum,* and *Thymus vulgaris* are effective against *Fusarium oxysporum* [105]. In addition to the antifungal effect, it is important that a product developed also has a low toxic effect on non-target organisms and the environment. In this sense, eugenol formulations were able to reduce ecotoxicity as well as maintain levels of cell viability without causing damage to DNA, suggesting that they are safe for the farmer as well as the crop [106].

Another strategy that has been adopted is the development of nanoparticles containing both plant extracts and ionic compounds. These systems have potentiated antimicrobial action against various phytopathogens [107]. In this context, silver nanoparticles loaded with an extract of *Tagetes patula* were effective against *Colletotrichum chlorophyti* under both laboratory and field conditions; that is, they inhibited the growth of a phytopathogen without affecting its host *Chlorophytum borivilianum* [108].

Similar to chemical substances that have physical-chemical characteristics that interfere with their application in nature, microorganisms used for biological control also suffer interference from biotic and abiotic factors. The encapsulation of microorganisms has been performed to enhance their effectiveness and protect them from damage caused by external factors. For example, microparticles containing *Trichoderma harzianum* were able to increase its action against *Sclerotinia sclerotiorum* 20-fold, improve its resistance to ultraviolet radiation, and control the release/growth of the microorganism [109].

Although formulations have shown the numerous advantages of the use of natural products and/or microorganisms with fungicidal action, so far, no data in the literature have reported the development of products with activity against *L. gongylophorus*. Thus, future studies on the encapsulation of the compounds and microorganisms highlighted in this review would be of great value in advancing new alternatives to suppress the fungus garden and, consequently, control the leaf-cutting ants.

## 4. Conclusions

Although insecticides have been used to control leaf-cutting ants, problems such as non-selective toxicity and environmental contamination justify the search for new alternatives. The symbiotic relationship with *L. gongylophorus* is essential for the maintenance of the nest, and substances that inhibit its growth are considered promising. Extracts obtained from *A. argentina*, *F. oolepis,* and *P. alopecuroides* species have shown relevant results against this symbiotic fungus. Isolated compounds such as argentilactone, xanthyletin, and platydesmine are examples of lactone, coumarin, and alkaloid, respectively, and would be the main classes responsible for the antifungal activity against *L. gongylophorus*. In addition, the synthesis of compounds would be an important alternative since structural modifications can increase the solubility of the compounds as well as potentiating their antifungal action. The development of formulations using micro- and nano-encapsulation technologies is another strategy to enable the use of natural compounds since they generally show low water solubility and low stability. Finally, the standardization of a method to evaluate the potential against *L. gongylophorus* is necessary to facilitate the analysis of results and the selection of the most promising compounds for commercial application.

## Data Availability

Not applicable.
